# A Short Period of Ventilation without Perfusion Seems to Reduce Atelectasis without Harming the Lungs during *Ex Vivo* Lung Perfusion

**DOI:** 10.1155/2013/729286

**Published:** 2013-09-11

**Authors:** Sandra Lindstedt, Leif Pierre, Richard Ingemansson

**Affiliations:** Department of Cardiothoracic Surgery, Skåne University Hospital, Lund University, 221 85 Lund, Sweden

## Abstract

To evaluate the lung function of donors after circulatory deaths (DCDs), *ex vivo* lung perfusion (EVLP) has been shown to be a valuable method. We present modified EVLP where lung atelectasis is removed, while the lung perfusion is temporarily shut down. Twelve pigs were randomized into two groups: modified EVLP and conventional EVLP. When the lungs had reached 37°C in the EVLP circuit, lung perfusion was temporarily shut down in the modified EVLP group, and positive end-expiratory pressure (PEEP) was increased to 10 cm H_2_O for 10 minutes. In the conventional EVLP group, PEEP was increased to 10 cm H_2_O for 10 minutes with unchanged lung perfusion. In the modified EVLP group, the arterial oxygen partial pressure (PaO_2_) was 18.5 ± 7.0 kPa before and 64.5 ± 6.0 kPa after the maneuver (*P* < 0.001). In the conventional EVLP group, the PaO_2_ was 16.8 ± 3.1 kPa and 46.8 ± 2.7 kPa after the maneuver (*P* < 0.01; *P* < 0.01). In the modified EVLP group, the pulmonary graft weight was unchanged, while in the conventional EVLP group, the pulmonary graft weight was significantly increased. Modified EVLP with normoventilation of the lungs without ongoing lung perfusion for 10 minutes may eliminate atelectasis almost completely without harming the lungs.

## 1. Introduction

Lung transplantation continues to be hampered by the number of available donors [1, 2]. *Ex vivo* lung perfusion (EVLP) has emerged as an essential tool for the reassessment, under a controlled scenario, of lungs from heart-beating donors (HBDs) that initially did not meet transplantation criteria [3–8]. The method is also an excellent tool for reassessing lungs of donors after cardiac death (DCD) [9, 10].

The use of DCD lungs has gained much interest lately. DCDs are classified according to the Maastricht classification and may be subdivided as controlled and uncontrolled [11]. Often the controlled DCDs are of interest since these patients are under hospital care, and their clinical history and lung function are known. It is also logistically easier to handle these donors. These controlled donors are, however, limited in number compared with the potential numbers of uncontrolled DCDs. The disadvantage of using lungs from uncontrolled donors, however, is that lung function is not known and has to be validated before the lungs can be accepted for transplant.

There are also some issues regarding the optimal preservation of uncontrolled donor lungs such as how long warm ischemic time the lungs can withstand and whether it is better to harvest the lungs after the period of warm ischemia or cool the lungs inside the deceased body. These issues are relevant for the donation team. The formalities of the donation process have to be also managed properly according to the law of each country.

As mentioned above, EVLP is also an excellent tool for reassessing DCD lungs. How to perform the optimal EVLP has also been a focus of the discussion. After the warming phase in EVLP, the lung is 37°C and fully ventilated, and it is ready to be reassessed for transplant suitability. To reassess the lungs adequately, all of the atelectasis has to be eliminated. In conventional EVLP, the atelectasis is eliminated by increasing the PEEP up to 10 cm H_2_O for 10–15 minutes at the time when the lungs have reached 37°C. The maneuver is performed, while the lung is maximally perfused. All of the lung atelectasis has to be eliminated for the lung to be tested adequately. If the lung is tested with the lung atelectasis present, a shunt will be formed where the blood is not oxygenated. In the present study, we hypothesize that elimination of atelectasis is preferably made under conditions where, the lung is ventilated but not perfused. In the present study the lung perfusion is temporarily shut down during 10 minutes. During that time, the lung is fully ventilated, and the PEEP is increased to 10 cm H_2_O. According to our knowledge, no such study has been performed previously.

## 2. Material and Methods

### 2.1. Animal Preparation

Twelve Swedish landrace pigs were fasted overnight with free access to water. The study was approved by the Ethics Committee for Animal Research, Lund University, Sweden (no. M 172-11). All animals received care according to the European Convention for the Protection of Vertebrate Animals Used for Experimental and Other Scientific Purposes, to the USA Principles of Laboratory Animal Care of the National Society for Medical Research, and to the Guide for the Care and Use of Laboratory Animals.

Premedication was performed with an intramuscular injection of xylazine (Rompun Vet. 20 mg/mL, Bayer AG, Leverkusen, Germany, 2 mg/kg) mixed with ketamine (Ketaminol Vet. 100 mg/mL, Farmaceutici Gellini S.P.A., Aprilia, Italy, 20 mg/kg) while the pig was still in its stable. Peripheral i.v. access was then established in the ear. The pig was then transferred to the laboratory and placed on the operating table in the supine position. Oral intubation was performed using a 7.5 mm endotracheal tube after the induction of anesthesia with sodium thiopental (Pentothal, Abbott Laboratories, North Chicago, IL, USA) and pancuronium bromide (Pavulon, N.V. Organon, Oss, the Netherlands). Anesthesia was maintained by infusions of ketamine (Ketaminol Vet.), midazolam (Midazolam Panpharma, Oslo, Norway), and fentanyl (Leptanal, Lilly, France). Fluid loss was compensated for by continuous infusion of Ringer's acetate. Mechanical ventilation was established with a Siemens-Elema ventilator (Servo Ventilator 300, Siemens, Solna, Sweden).

### 2.2. Preservation of the Lungs

Ventricular fibrillation was induced electrically. The tracheal tube was disconnected from the ventilator when circulatory arrest was confirmed. The animals were left untouched for 1.5 hours at room temperature. Thereafter, a median sternotomy was performed. The pulmonary artery was cannulated via the right ventricle with a 28 F cannula secured with a purse string suture placed in the outflow tract of the A. pulmonalis. A clamp was placed on the v. cava superior, and another clamp on the v. cava inferior. A third clamp was then placed on the ascending aorta. The left atrial and the v. cava inferior were then opened. The right and left pleurae were filled with ice slush to cool the lungs.

The lungs were perfused antegradely with 5 L of cold Perfadex containing 1.0 mL isotonic trometamol (Addex-THAM 3.3 mmol/mL, Fresenius Kabi AB, Uppsala, Sweden), 2 mL calcium chloride (0.45 mmol/mL), and 3 mL nitroglycerine (5 mg/mL, BMM Pharma AB, Stockholm, Sweden) at a low perfusion pressure (<20 mmHg). The cannula was then removed from the pulmonary artery. The lungs were harvested en bloc in a standard fashion and weighed. A segment (*∼*8 cm) of the descending aorta was also excised. The lungs, together with the aortic segment, were then immersed in cold Perfadex and kept in cold storage at 6°C for 2 hours.

### 2.3. *Ex Vivo* Lung Perfusion

EVLP was performed using the Medtronic *Ex Vivo* Lung Evaluation Set extracorporeal perfusion Circuit (Medtronic AB, Kerkrade, the Netherlands; *Ex Vivo* Lung Evaluation Set). The system was primed with albumin (500 mL, 50 g/L, and 200 mL 200 g/L; Albumin Baxter, Baxter Medical, Kista, Sweden) and 2 units of autologous blood, withdrawn previously from each donor. Imipenem (0.5 g, Tienam, Merck Sharp & Dohme, Sollentuna, Sweden), insulin (20 IU, Actrapid, Novo Nordisk, Bagsvaerd, Denmark), and heparin (10,000 IU, Leo Pharma, Malmö, Sweden) were added, and isotonic trometamol (Addex-THAM, Kabi, Sweden) was used to buffer the mixed solution to a temperature-adjusted pH of 7.4.

Gas was supplied to the affinity membrane oxygenator (Medtronic, Minneapolis, NJ, USA): first oxygen and CO_2_ during the reconditioning phase and then 93% nitrogen and 7% CO_2_ during the testing phase, creating a normal venous blood gas in the perfusate to the pulmonary artery (in other words, the oxygenator was used to deoxygenate the perfusate). Before starting perfusion, the pulmonary artery was extended using the excised segment of the descending aorta to facilitate cannulation. The pulmonary artery cannula was then connected to the corresponding tube of the extracorporeal circuit, the air was removed, and the shunt of the circuit was clamped. An endotracheal tube was secured in the trachea with a cotton band and connected to the ventilator. The remnant of the left atrium was left open, preventing obstruction of the pulmonary outflow since the perfusion solution flowed directly out into the lung reconditioning box. The left atrium pressure was, therefore, 0 mmHg.

Low-flow perfusion at 25°C was initiated through the lungs. The lungs were gradually warmed by increasing the temperature of the perfusate. When the temperature reached 32°C, ventilation was started with an inspired oxygen fraction of 0.5 and a minute volume of 1 L/minute, with no PEEP. The pump flow was gradually increased, but the pulmonary arterial pressure was never allowed to exceed 20 mmHg. With each 1°C increase in temperature, the ventilation was increased by a minute volume of 1 L. When the perfusate from the lung reached 37°C, normal ventilation (100 mL · kg^−1^ · min⁡^−1^) was given, but with no PEEP. The lungs were then randomly assigned to one of the two groups: one receiving conventional EVLP and one receiving modified EVLP with the intention of eliminating lung atelectasis according to the following protocol.

### 2.4. Conventional EVLP

In the conventional EVLP group, the lung atelectasis was eliminated by temporarily increasing PEEP to 10 cm H_2_O for 10 minutes when the perfusate from the lung reached 37°C. The perfusion through the lung (pulmonary artery flow) was kept unchanged during this time. The PEEP was then removed, and the lung was ventilated (100 mL · kg^−1^ · min⁡^−1^) for 5 minutes without PEEP at an inspired oxygen fraction of 1.0. Blood gases were then analyzed during perfusion and ventilation (100 mL · kg^−1^ · min⁡^−1^), with PEEP being 5 cm H_2_O at an inspired oxygen fraction of 1.0. The lungs were then disconnected from the EVLP equipment. The lungs were then weighed once again.

### 2.5. Modified EVLP

In the modified EVLP group, the lung perfusion was shut down (e.g., stopping the circulation) when the perfusate from the lung reached 37°C. During the shutdown time of 10 minutes, the ventilation was kept at 100 mL · kg^−1^ · min⁡^−1^, and PEEP was increased to 10 cm H_2_O, creating a system where the lungs are fully ventilated without any perfusion. The system was not disconnected during the shutdown time to eliminate the risk of air entering the system. The PEEP was then removed, the perfusion was reestablished, and the lung was ventilated at 100 mL · kg^−1^ · min⁡^−1^ for 5 minutes without PEEP. Blood gases were then analyzed during perfusion and ventilation (100 mL · kg^−1^ · min⁡^−1^), with PEEP being 5 cm H_2_O at an inspired oxygen fraction of 1.0. The lungs were then disconnected from the EVLP equipment. The lungs were then weighed once again.

### 2.6. Calculations and Statistics

Calculations and statistical analysis were performed using GraphPad 4.0 software. Statistical analysis was performed using one-way ANOVA and Bonferroni's multiple comparison test, comparing all groups. A level of *P* < 0.05 was considered statistically significant, and *P* > 0.05 was considered not significant (n.s.). The results are presented as median and range or mean and standard error of the mean (SEM).

## 3. Results

### 3.1. Study Groups

No significant differences were observed in animal weight in the two groups (72 ± 1 kg in the modified EVLP group and 73 ± 2 kg in the conventional EVLP group (*P* > 0.05)). Neither were there any differences in arterial oxygen partial pressure at an inspired oxygen fraction of 1.0 (64.8 ± 6.0 kPa in the modified EVLP group and 67.7 ± 1.8 kPa in the conventional EVLP group) before organ harvesting. No anatomical anomalies, signs of infection, or malignancies were found in any of the animals at autopsy.

### 3.2. The *Ex Vivo* Lung Perfusion

The time from the initiating of the EVLP circuit and until the perfusate from the lung had reached 37°C was calculated. No significant differences in the EVLP time were observed in the two groups (25 ± 2 minutes in the modified EVLP group and 26 ± 3 minutes in the conventional EVLP group (*P* < 0.05)). After the perfusate from the lungs had reached 37°C, the lungs were randomly assigned to one of the two groups: one receiving conventional EVLP and one receiving modified EVLP with the intention of eliminating lung atelectasis. The time from when the perfusate from the lung had reached 37°C until completing EVLP (total EVLP time) was calculated. No significant differences in total EVLP time were observed in the two groups (40 ± 3.2 minutes in the modified EVLP group and 41 ± 2.9 minutes in the conventional EVLP group (*P* < 0.05)).

### 3.3. Pulmonary Graft Function

#### 3.3.1. Arterial and Venous Blood Gases

**Figure 1:**
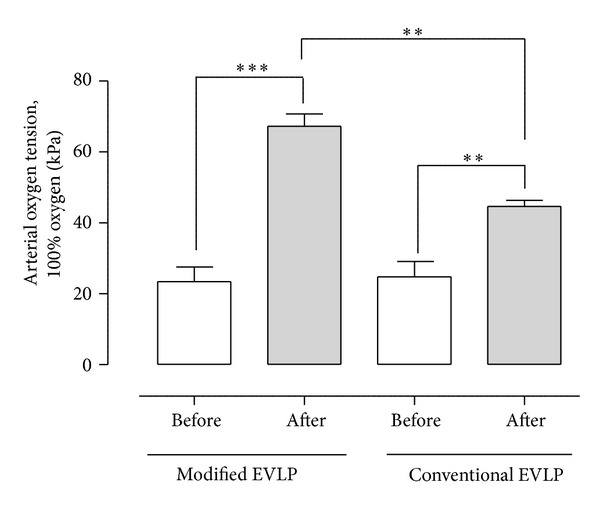
Mean arterial oxygen tension (± SEM) before and after modified and conventional EVLP in the aspect of eliminating lung atelectasis after initial EVLP where the lung is warmed up to 37°C and ventilated according to the golden standard for EVLP. Statistical analysis was performed using ANOVA. *N* = 6 in each group. Significance was defined as *P* < 0.05 (*), *P* < 0.01 (**), *P* < 0.001 (***), and *P* > 0.05 (not significant, n.s.).

Samples of blood were taken before and after passing through the lungs (i.e., venous and arterial blood samples) to measure blood gases after 5-minutes exposure to fractions of inspired oxygen (FiO_2_) at 1.0. The arterial blood gases and the venous blood gases after 5 minutes of ventilation with FiO_2_ at 1.0 are presented in Table 1. Notice the significant increase in arterial blood gases in both groups after performing one of the maneuvers for eliminating the lung atelectasis. In the modified EVLP group, the arterial oxygen partial pressure (PaO_2_) was 18.5 ± 7.0 kPa before the maneuver and 64.5 ± 6.0 kPa after the maneuver (*P* < 0.001). In the conventional EVLP group, the PaO_2_ was 16.8 ± 3.1 kPa before the maneuver and 46.8 ± 2.7 kPa after the maneuver (*P* < 0.01). Comparing the two groups, the modified EVLP group showed significantly improved PaO_2_ after the maneuver compared with the conventional EVLP group (*P* < 0.01) (Figure 1).

#### 3.3.2. Airway Pressure

**Figure 2:**
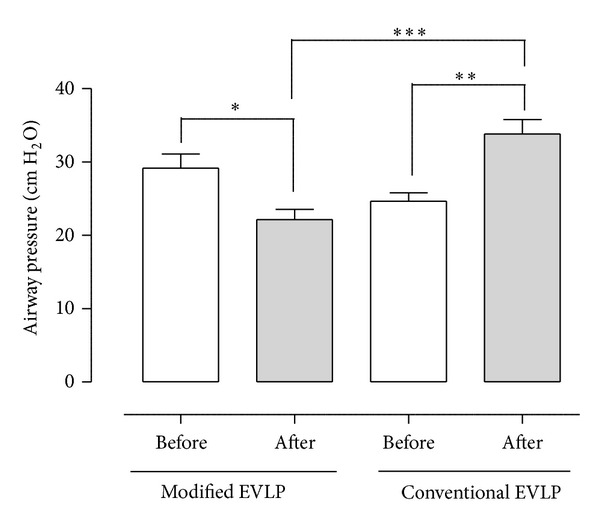
Mean airway pressure (± SEM) before and after modified and conventional EVLP in the aspect of eliminating lung atelectasis after initial EVLP where the lung is warmed up to 37°C. Statistical analysis was performed using ANOVA. *N* = 6 in each group. Significance was defined as *P* < 0.05 (*), *P* < 0.01 (**), *P* < 0.001 (***), and *P* > 0.05 (not significant, n.s.).

The airway pressure (AP) is also presented in Table 1. Note the significant decrease in AP in the modified EVLP group before (27.0 ± 2.9 cm H_2_O) and after (20.0 ± 2.0 cm H_2_O) the maneuver (*P* < 0.05). Note also the increase in AP in the conventional EVLP group before (24.3 ± 1.5 cm H_2_O) and after (30.7 ± 1.8 cm H_2_O) the maneuver (*P* < 0.01). Comparing the two groups, the modified EVLP group showed lower AP after the maneuver compared with the conventional EVLP group (*P* < 0.001) (Figure 2).

#### 3.3.3. Pulmonary Vascular Resistance

Pulmonary vascular resistance (PVR) was calculated using the following formula: PVR (dyne × s/cm^5^) = [(80 ∗ PAP) – LAP]/CO, where PAP is the mean pulmonary artery pressure, LAP is the left atrium pressure, and CO is the cardiac output (CO is equivalent to pulmonary artery flow in the EVLP method).

**Figure 3:**
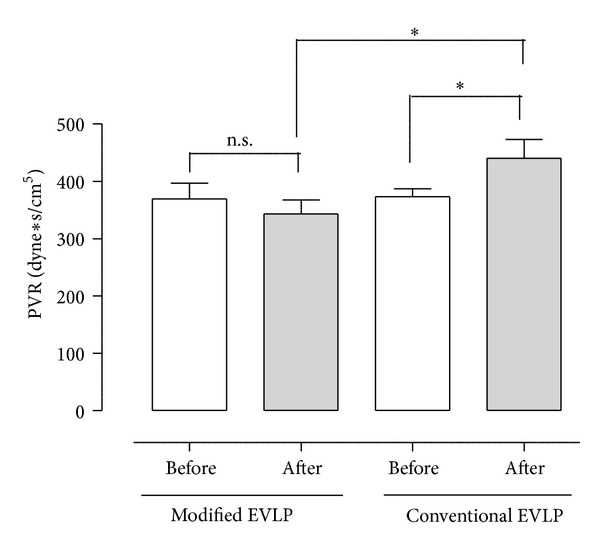
Mean pulmonary vascular resistance (PVR) (± SEM) before and after modified and conventional EVLP in the aspect of eliminating lung atelectasis after initial EVLP where the lung is warmed up to 37°C and ventilated according to the golden standard for EVLP. Statistical analysis was performed using ANOVA.  *N* = 6  in each group. Significance was defined as *P* < 0.05  (*),  *P* < 0.01  (**),  *P* < 0.001  (***), and  *P* > 0.05  (not significant, n.s.).

The pulmonary vascular resistance was calculated after ventilation with FiO_2_ at 1.0 and is also presented in Table 1. In the modified EVLP group, the PVR was 359 ± 57 before the maneuver and 333 ± 48 after the maneuver, a slight but nonsignificant decrease (*P* > 0.05). In the conventional EVLP group, the PVR was 366 ± 24 before the maneuver and 464 ± 39 after the maneuver (*P* < 0.05). Comparing the two groups, the modified EVLP group showed significantly lower PVR after the maneuver compared with the conventional EVLP group (*P* < 0.05) (Figure 3).

#### 3.3.4. Pulmonary Graft Weight

The lungs were weighed after harvesting and after EVLP to assess the degree of  lung edema.

The time from disconnecting the EVLP circuit and until the lungs were weighed was calculated. No significant differences in the EVLP time were observed in the two groups (3 ± 0.3 minutes in the modified EVLP group and 3 ± 0.5 minutes in the conventional EVLP group (n.s.)).

**Figure 4:**
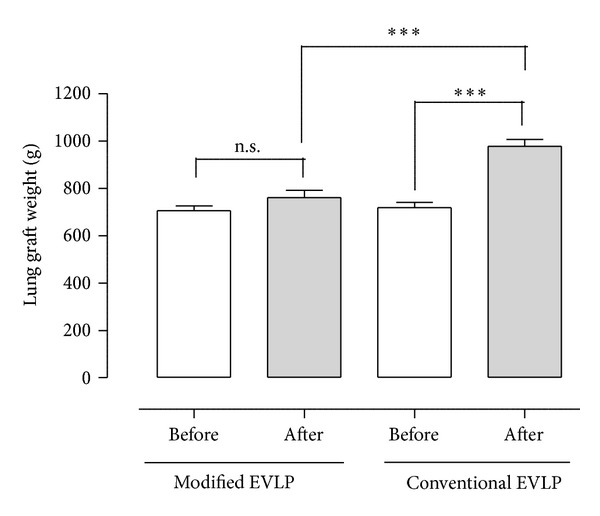
Mean lung weight (± SEM) before and after modified and conventional EVLP in the aspect of eliminating lung atelectasis after initial EVLP where the lung is warmed up to 37°C and ventilated according to the golden standard for EVLP. Statistical analysis was performed using ANOVA.  *N* = 6  in each group. Significance was defined as  *P* < 0.05 (*),  *P* < 0.01  (**),  *P* < 0.001  (***), and  *P* > 0.05  (not significant, n.s.).

The results are shown in Figure 4. Interestingly, the pulmonary grafts receiving the modified EVLP showed unchanged weight before and after the maneuver, indicating a dry pulmonary graft with good quality, while the pulmonary grafts from the conventional EVLP showed increased pulmonary graft weight indicating a wet and heavy pulmonary graft with poor quality.

In the modified EVLP group, the pulmonary graft weight was  723 ± 43  grams before the maneuver and 746 ± 43 grams after the maneuver (n.s.). In the conventional EVLP group, the pulmonary graft weight was 690 ± 15 grams before the maneuver and 973 ± 60 grams after the maneuver (*P* < 0.001). Comparing the two groups, the modified EVLP group showed significantly lower pulmonary graft weight after the maneuver compared with the conventional EVLP group (*P* < 0.001) (Figure 4).

### 3.4. Hemodynamic Data

Pulmonary artery flow (L/min), that is, cardiac output (CO), in the *ex vivo* model and pulmonary artery pressure (PAP) were measured continuously. The pulmonary artery flow was not allowed to exceed 4 L/min, and the PAP was not allowed to exceed 20 mmHg. No significant differences in pulmonary artery flow or PAP were seen before and after the different maneuvers in the two groups or between the two groups during the evaluation of the pulmonary graft. The data are presented in Table 1.

### 3.5. Macroscopic Appearance

After completing the lung evaluation, the pulmonary arterial branches were macroscopically studied for thrombotic material by opening the arteries distally as far as possible. No thrombotic material was observed in either of the groups.

## 4. Comment

Lung transplantation is used to treat patients with a variety of end-stage pulmonary diseases [12]. While indications for lung transplantation continue to increase, widespread application of this procedure remains limited due to the lack of suitable donor organs [7]. Lately, great interest has been focused on the expansion of the donor pool using organs from DCDs [7, 9, 13, 14]. There are both ethical and medical problems associated with this.

Some years ago, we reported the results of the first six double-lung transplantations performed in the world with donor lungs from HBDs that were rejected for transplantation by the Scandiatransplant, the Eurotransplant, and the UK Transplant organizations after *in situ* evaluation due to poor arterial oxygen tension [15]. The EVLP method, which has been described in detail previously [5, 15, 16], has been suggested as a novel method of differentiating between “good” and “poor” pulmonary grafts in a group referred to as marginal donors due to poor arterial oxygen tension. We found EVLP to be an excellent tool to reveal lung pathology and to evaluate lung function prior to decision making regarding lung transplantation, thereby increasing the number of potential lungs available for transplantation. EVLP has also been successful in evaluating lung function in experimental lung preservation and lung transplantation [6, 17]. When grafts characterized as good using this method were transplanted, the results revealed no significant differences from those with lungs fulfilling the standard criteria [7]. However, these patients often showed a slight increase in primary graft dysfunction, displayed as slight increase in interstitial whitening, on the chest X-ray on the first days after the transplant.

In the present study, we present a modified form of EVLP for eliminating atelectasis. In conventional EVLP, the golden standard to eliminate the atelectasis is to increase PEEP up to 10 cm H_2_O for 10–15 minutes after the lung has reached 37°C. The perfusion of the lung is kept on maximum flow, while the lungs are ventilated with increased PEEP. We have noticed in earlier studies (work in progress) that during this maneuver it is very easy to harm the lung and create a slight edema while eliminating the atelectasis. However, it is crucial to eliminate the atelectasis to be able to perform an adequate validation of the lungs. If there is atelectasis, a shunt will be formed in this part of the lung resulting in nonfunctional lung parenchyma. This may lead to false low blood gas values and, in the worst case, failure to meet the criteria for lung transplantation. In the present study, we hypothesize that lung injury could be minimized by shutting down the perfusion without disconnecting the EVLP circuit, ventilating the lung with increased PEEP for 10 minutes. We refer to this maneuver as modified EVLP. As expected, both groups had significant higher blood gases after eliminating the atelectasis, but the lungs from the modified EVLP group had significant higher blood gases compared with the conventional EVLP group. Both groups, however, met the criteria for acceptance for lung transplantation. Interestingly, the lungs receiving modified EVLP had a significant lower lung weight compared with the conventional EVLP lungs indicating less lung injury and lung edema with the modified EVLP. There were also significantly higher airway pressure and PVR in the conventional EVLP group compared with the modified EVLP group, also indicating lung injury in the conventional EVLP group. Clinically, it is preferable to transplant a dry lung compared with a slightly heavier lung due to interstitial lung edema. The recipient who receives the dry lung with less lung injury will likely experience shorter time on a ventilator and shorter time at the ICU after the transplantation. The signs of primary graft dysfunction will probably be also less, which will affect the recipient's morbidity and longtime mortality. Limitations in our study are the rather small study groups, the use of the EVLP and not a transplant model, and being assessed *in vivo*. The observations from our study need to be taken into a clinical setting to be further assessed.

## 5. Conclusions

Modified EVLP with normoventilation of the lungs without ongoing lung perfusion for 10 minutes may eliminate atelectasis almost completely without harming the lungs, thereby making it possible to evaluate blood gases properly in order to decide whether to transplant.

## Figures and Tables

**Table 1:** Samples of blood were taken before and after passing through the lungs (i.e., venous and arterial blood samples) to measure blood gases after 5-minute exposure to fractions of inspired oxygen (FiO_2_) at 1.0.

	Before & after modified EVLP			Before & after conventional EVLP			Total
	Before	After	*P* value	Before	After	*P* value	*P* value
FiO_2_ 1.0							
PAP (mmHg)	17 ± 2.1	16.7 ± 2.4	n.s.	18 ± 1.2	20 ± 0.0	n.s.	n.s.
CO (L/min)	3.8 ± 0.2	4.0 ± 0.0	n.s.	3.9 ± 0.1	3.5 ± 0.3	n.s.	n.s.
AP (cm H_2_O)	27 ± 2.9	20 ± 2.0	**<0.05**	24.3 ± 1.5	30.7 ± 1.8	**<0.01**	**<0.001**
PvCO_2_ (kPa)	4.2 ± 0.3	4.4 ± 0.2	n.s.	4.0 ± 0.2	4.0 ± 0.1	n.s.	n.s.
PvO_2_ (kPa)	7.5 ± 0.4	8.0 ± 0.5	n.s.	7.6 ± 0.7	7.5 ± 0.3	n.s.	n.s.
PaCO_2_ (kPa)	3.9 ± 0.1	3.7 ± 0.1	n.s.	4.0 ± 0.1	3.8 ± 0.1	n.s.	n.s.
PaO_2_ (kPa)	18.5 ± 7	64.5 ± 6	**<0.001**	16.8 ± 3.1	46.8 ± 2.7	**<0.01**	**<0.01**
PVR ((dynes × s)/cm^5^)	359 ± 57	333 ± 48	**n.s.**	366 ± 24	464 ± 39	**<0.05**	**<0.05**
Weight (gram)	723 ± 43	746 ± 43	**n.s.**	690 ± 15	973 ± 60	**<0.001**	**<0.001**

FiO_2_: inspired oxygen fraction, PAP: pulmonary arterial pressure, CO: cardiac output/pulmonary artery flow, AP: mean airway pressure, PvCO_2_: venous carbon dioxide partial pressure, PvO_2_: venous oxygen partial pressure, PaCO_2_: arterial carbon dioxide partial pressure, PaO_2_: arterial oxygen partial pressure, and PVR: pulmonary vascular resistance.
